# Quality of life in children newly diagnosed with cancer and their mothers

**DOI:** 10.1186/1477-7525-3-29

**Published:** 2005-04-28

**Authors:** Christine Eiser, J Richard Eiser, Christopher B Stride

**Affiliations:** 1 CR-UK Child and Family Research Group, Department of Psychology, University of Sheffield S10 2TP, UK; 2 Institute of Work Psychology, Department of Psychology, University of Sheffield, S10 2TP, UK

**Keywords:** Childhood, Cancer, Quality of life, proxy ratings.

## Abstract

**Background:**

With current treatments, approximately 75% of children diagnosed with cancer can expect to achieve disease-free survival. However, treatments are complex and aggressive, potentially compromising QOL for children and their parents. Although previous work has shown increased anxiety and depression among parents after diagnosis, the recent development of standardised measures of QOL enables us to look more directly at the impact of diagnosis on mothers' and children's QOL. The aims of this study are to i) describe QOL for children and their mothers after diagnosis by comparing their scores with population norms, ii) explore the relationship between mothers' worries about the illness and their QOL, and iii) determine the relationship between mothers ratings of their own QOL and their child.

**Method:**

A total of 87 families took part, constituting 60% of those eligible. The children included 58 males and 29 females aged between 2 years 6 months to 16 years 3 months (mean = 7 years, median = 5 years 8 months). Diagnoses were acute lymphoblastic leukaemia (ALL, n = 57), brain tumours (n = 11), bone tumours (n = 17) and 2 rare cancers. Mothers completed questionnaires about their own and the child's QOL.

**Results:**

Mothers' reported their own and the child's QOL to be significantly lower than population norms. There were significant correlations between mothers' worries and their own and their ratings of the child's QOL and mothers' ratings of their own QOL correlated with their ratings of the child's QOL.

**Conclusion:**

Both children and their mothers experience significantly compromised QOL in the months following diagnosis. Mothers who rated their own QOL to be poor also rate their child's QOL to be low. These results suggest caution is required where mothers rate their child's QOL. Efforts must continue to be made to improve QOL of children especially in the period immediately following diagnosis.

## Background

Survival in childhood cancer has improved significantly in recent years [1,2]. This has been attributed to the organisation of care in key centres, and development of national and international clinical trials which facilitate more rapid knowledge and refinements of new protocols. Depending on the specific cancer, children are treated with a combination of radiotherapy, chemotherapy and surgery. Duration of chemotherapy also varies but can be up to 3 years for boys treated for the most common cancer, acute lymphoblastic leukaemia (ALL).

Despite the improved survival statistics, cancer remains a potentially life-threatening condition, and as such poses a major challenge to both child and family. During the course of treatment, most children experience unpleasant physical side-effects. Behavioural and emotional problems have also been identified. In the longer term, there is a considerable risk of late effects [3]. These include reduced linear growth, compromised endocrine and sensory functions, and damage to cardiac and reproductive systems.

In addition to the effects on children, adverse consequences for parents' immediate and longer-term physical and mental health have been reported [4-6]. Many parents report elevated levels of depression and anxiety especially in the months immediately after diagnosis [7], but for most this decreases over time [5,8,9].

Improvements in survival are the result of increasingly aggressive treatments, raising questions about the *quality *of life (QOL) as well as *quantity *or length of survival. For the child, QOL is likely to be compromised by the pain of illness and treatment, lack of energy to enjoy everyday activities, and fears about the future. Mothers themselves experience great changes to their lives, staying in hospital with their child, perhaps giving up or reducing the hours they spend at work, as well as learning how to manage the child's medical care at home. The relatively recent emergence of standardised QOL measures provides the opportunity to formalise the extent to which QOL is compromised for mothers and children following diagnosis, and to describe any differences in impact depending on characteristics of the child (age, gender) and illness.

In this study, we obtained ratings from mothers about their own, and their child's QOL, and compared these with population norms. We predicted that, in the months immediately after diagnosis, mothers would rate their own and their child's QOL significantly below norms for the normal population. Second, we explored relationships between mothers' QOL and more illness-specific worries, and third, predicted that mothers who rated their own QOL to be poor would also rate their child's QOL to be poor.

## Methods

### Procedure

Families of a newly diagnosed child were invited to participate in a study about coping with the child's illness. Families were approached approximately three months after diagnosis, as the child's condition is normally relatively stable, and treatment is on an out-patient basis. Families were approached by the clinic nurse at a routine clinic visit and given written and verbal information about the study. Those who agreed to participate gave signed consent and were subsequently contacted by the research team who visited the family at home. All aspects of the procedure were approved by the Hospital Ethics Committees.

### Sample

English-speaking families of children aged 2–18 years diagnosed at five cancer centres in the UK over a two-year period were approached. Exclusions were children with advanced disease, cognitive or neurological impairment prior to diagnosis, or other complicating conditions (e.g. Down's syndrome).

A total of 87 families took part (60%) of those eligible. Families who refused cited lack of interest, too distressed or too busy as explanations. The sample was predominantly Caucasian; there were two families of Asian origin, but both had lived in the UK for more than 10 years. There were 58 males and 29 females. The ages of the children ranged from 2 years 6 months to 16 years 3 months (mean = 7 years, median = 5 years 8 months). Children were being treated for acute lymphoblastic leukaemia (ALL, n = 57), brain tumours (n = 11), bone tumours (n = 17) and 2 rare cancers.

### Questionnaires

#### Child's QOL

The Pediatric Quality of Life Inventory (PedsQL ™ 4.0) [10] includes subscales to measure physical health, social, school and emotional functioning. There are separate age-appropriate versions including one for children aged 2.5–4 years. For each item, mothers are asked how much of a problem has been experienced over the last month. Items are rated on 5-point Likert scales, from 0 (never a problem) to 4 (almost always a problem). After aggregation and transformation, subscale scores range from 0–100, with higher scores representing better QOL.

The Maternal worry scale [11] is an 11-item scale that measures mothers' worries concerning the child's future. Examples of the items include worries about future reliance on medication, becoming worse in the future and feeling different from others as a result of the illness. The scale has shown adequate psychometric properties, an internal consistency (Cronbach's α) of 0.94 and a test-retest reliability of 0.84.

#### Mothers' well-being

The SF-36 scale [12] was included as a measure of mothers' own well-being. This includes a single-item measure of change in health, plus eight subscales, with varying numbers of items and response formats, defined as physical functioning, role limitation (physical), role limitation (social), social functioning, mental health, energy/vitality, pain and general health perception. The scale has been extensively used in health services research, has established psychometric properties and UK norms [13].

#### Medical data

Information about diagnosis and date of diagnosis were obtained from medical records.

### Treatment of results

There was considerable missing data for the school functioning subscale of the PedsQL ™ 4.0, which mothers did not complete whenever children were below school age or had not yet returned to school after diagnosis. This subscale was therefore excluded from analyses. We then calculated reliabilities for all the remaining scales and subscales. Internal consistency was consistently good (see Table 1). Comparisons between sample means and population means were based on 1 sample t-tests and relationships between variables investigated suing simple correlations. All analyses were conducted using SPSS version 10 for Macintosh.

**Table 1:** Child and parental QOL measures: means, norms and reliabilities

**Measure**	**(n)**	**mean**	**Norm**	**alpha**	**t**
**Child's QOL**					
Physical Functioning	74	36.6*	84.9	0.86	-20.2
Emotional Functioning	74	48.2*	74.7	0.75	-10.76
Social Functioning	74	66.4*	86.6	0.80	-7.63
**Parent's QOL**					
Physical Function	66	91.77	89.5	0.91	1.19
Social Functioning	68	59.2*	86.9	0.82	-7.37
Role Limitation – Physical	69	67.0*	84.6	0.85	-3.82
Role Limitation – Emotional	66	47.0*	80.6	0.77	-6.48
Mental Health	69	54.5*	72.0	0.76	-7.16
Energy/Vitality	68	44.6*	58.6	0.80	-5.01
Pain	68	84.2	79.9	0.77	1.48
General Health Perception	68	73.0	75.0	0.85	-0.86
Worry	65	2.07	N/A	0.87	

* Sample mean indicating significantly lower QOL than norm (*p *< .05, 1-tailed test).

## Results

### Comparisons with population norms

Consistent with our first hypothesis, mothers reported significantly lower QOL for their child compared with population norms (14). They also reported their own QOL to be lower than expected across most subscales (13), notable exceptions being the SF-36 subscales relating to physical function and pain, where mothers reported functioning within the normal range. These data are shown in Table 1.

### Relationship between mothers' own QOL and worries

Mothers who reported more worries rated their own (r = -.53) and the child's QOL (Physical r = -.31; Emotional r = -.36; Social r = -.42) to be lower.

### Relationship between mothers' own QOL and their ratings of the child's QOL

There were significant correlations between mothers' ratings of their own QOL and their ratings of the child's QOL (Physical: r = 0.43; Emotional r = 0.43; and Social r = 0.52). Mothers' ratings of the child's QOL suggested no differences depending on diagnosis, child age or gender.

## Discussion

Our results suggest that, in the three to five months following diagnosis of ALL, mothers report that children's QOL is significantly compromised compared with the normal population. Although this is to be expected, the extent to which QOL scores were below population means was considerable. On most subscales of the SF-36, mothers also reported their own QOL to be lower than population means. Our results attest to the huge burden experienced by children and their parents during the initial period of treatment for cancer.

Although correlations give no information about the direction of a relationship (whether worries compromised QOL or vice-vera), we found that mothers who were more worried reported lower QOL themselves. Finally, mothers' ratings of their own QOL were correlated with their ratings of the child's QOL. Although it is normally recommended that ratings of QOL should be made by the patient wherever possible, it has always been acknowledged that there are situations, especially where children are very young or ill, when it is necessary to rely on parents' ratings. Diagnosis of cancer in a child is likely to be one of those situations, in so far as children are often young, ill and distressed. Our data suggest that mothers can provide ratings of the child's QOL but these are related to their own QOL. Mothers who rated their own QOL to be lower and reported more worries, rated the child's QOL to be worse. There is a question about how well mothers are able to report their child's QOL. A number of reports suggest there are differences between mothers and children in their views of the child's QOL, although these have not been related in any simple way to variables such as age or gender [15].

Study limitations include reliance on mothers' ratings of their own and their child's QOL. Although we had wanted to measure children's QOL by asking them directly, there were a number of obstacles to achieving this. First, the average age on diagnosis of ALL is 4 years, meaning that many children were simply too young to respond for themselves. Second, on diagnosis, even the older children tended to be too ill to respond.

The reliance on mothers, rather than fathers, may also be a limitation. Mothers tend to be more involved with the care of the sick child, more responsible for medication and treatment decisions, and more likely to stay in hospital with the child. In contrast, fathers tend to work as much as possible and generally try to maintain normal family life for other children in the family [16]. This difference in experience, resulting in fathers remaining more involved in everyday life despite the child's illness, may have an impact on parenting and parents' perceptions of the child's QOL.

In addition, our sample included only 60% of those eligible. We have no way of knowing whether those who refused differed in any crucial way from families who agreed to take part, though typically they stated they were too busy or distressed. Given the suddenness of the diagnosis and the amount of hospitalisation and care typically needed, it is not surprising that some families were overwhelmed and did not wish to add an additional demand on their time. As Ethics committees do not allow investigators to probe families reasons for agreeing or not to take part in research, we are unable to be any more precise on this point.

A further limitation follows from use of PedsQL ™ 4.0 as a measure of QOL. The PedsQL ™ 4.0 was developed as a generic measure of children's QOL, and therefore does not tap specific implications associated with diagnosis and treatment. Mothers failed to complete ratings in the schools subscale, on the basis that many children were attending sporadically or experienced very lengthy absences. This suggests the measure may lack sensitivity for newly diagnosed children and challenges the adequacy of generic measures of QOL to provide a comprehensive assessment of QOL in children with serious illness. Use of cancer specific scales would be helpful [17], though currently lack UK norms.

It is important that work of this kind has clinical implications for parents and medical staff. There are concerns that the treatments currently used to treat cancer may unnecessarily compromise child QOL, both during treatment and in the longer term [18]. Although use of Hickman lines and anti-emetic drugs are now routine and have reduced children's experiences of pain and chemotherapy related sickness, our data suggest much still needs to be done to improve QOL in children on treatment. Fear of infection and children's fatigue mean that families lead very isolated lives after diagnosis. If QOL is to be further improved, we need to find ways to reduce the sense of loneliness and isolation experienced, as well as family fears for the future.

## Authors' contributions

C. Eiser conceived the study. JR Eiser and CB Stride were responsible for analyses, and all contributed to the write-up.

## References

[B1] Stiller CA (1994). Population based survival rates for childhood cancer in Britain, 1980–91. BMJ.

[B2] Stiller CA, Bunch K, Lewis I (2000). Ethnic group and survival from childhood cancer: Report from the UK Children's Cancer Study Group. Br J Cancer.

[B3] Wallace H, Green D (2004). Late effects of childhood cancer.

[B4] Manne S, Miller D, Meyers P, Wollner N, Steinherz P, Redd W (1996). Depressive symptoms among parents of newly diagnosed children with cancer: A 6-month follow-up study. Child Health Care.

[B5] Nelson A, Miles M, Reed S, Davis C, Cooper H (1994). Depressive symptomatology in parents of children with chronic oncologic or hematologic disease. J Psychosoc Oncol.

[B6] Vance YH, Morse R, Jenney M, Eiser C (2001). Issues in measuring quality of life in childhood cancer: Measures, proxies, and parental mental health. J Child Psychol Psychiatry.

[B7] Grootenhuis MA, Last BF (1997). Predictors of parental emotional adjustment to childhood cancer. Psycho-Oncology.

[B8] Dahlquist LM, Czyzewski, Copeland KG, Jones CL, Taub E, Vaughan JK (1993). Parents of children newly diagnosed with cancer: Anxiety, coping and marital distress. J Pediatr Psychol.

[B9] Sawyer M, Streiner D, Antoniou G, Toogood I, Rice M (1998). Influence of parental and family adjustment on later psychological adjustment of children treated for cancer. J Am Acad Child Adolesc Psychiatry.

[B10] Varni J, Seid M, Rode C (1999). The PedsQL: Measurement model for the pediatric quality of life inventory. Med Care.

[B11] DeVet K, Ireys H (1998). Psychometric properties of the maternal worries scale for children with chronic illness. J Pediatr Psychol.

[B12] Ware JE, Snow KK, Kosinski M, Gandek B (1993). SF-36 health survey manual and interpretation guide.

[B13] Jenkinson C, Coulter A, Wright L (1993). Short form 36 (SF-36) health survey questionnaire normative data for adults of working age. BMJ.

[B14] Upton P, Eiser C, Cheung I, Hutchings HA, Jenney M, Maddocks A, Russell IT, Williams JG (2005). Measurement properties of the UK-English version of the Pediatric Quality of Life Inventory 4.0 (PedsQL) generic core scales. Health Qual Life Outcomes.

[B15] Eiser C, Morse R (2001). Quality of life in chronic diseases of childhood. Health Technol Assess.

[B16] Wysocki T, Gavin L (2004). Psychometric properties of a new measure of fathers' involvement in the management of pediatric chronic diseases. J Pediatr Psychol.

[B17] Varni JW, Rode CA, Seid M, Katz ER, Friedman-Bender A, Quiggins DJL (1999). The pediatric cancer quality of life inventory-32 (PCQL-32). II. Feasibility and range of measurement. J Behav Med.

[B18] Craft AW (2000). Childhood cancer – mainly curable so where next?. Acta Paediatr.

